# Validating automated eye disease screening AI algorithm in community and in-hospital scenarios

**DOI:** 10.3389/fpubh.2022.944967

**Published:** 2022-07-22

**Authors:** Ruoan Han, Gangwei Cheng, Bilei Zhang, Jingyuan Yang, Mingzhen Yuan, Dalu Yang, Junde Wu, Junwei Liu, Chan Zhao, Youxin Chen, Yanwu Xu

**Affiliations:** ^1^Key Laboratory of Ocular Fundus Diseases, Department of Ophthalmology, Peking Union Medical College Hospital, Chinese Academy of Medical Sciences, Beijing, China; ^2^Intelligent Healthcare Unit, Baidu, Beijing, China

**Keywords:** glaucoma, macula, diabetic retinopathy, artificial intelligence, eye disease screening

## Abstract

**Purpose:**

To assess the accuracy and robustness of the AI algorithm for detecting referable diabetic retinopathy (RDR), referable macular diseases (RMD), and glaucoma suspect (GCS) from fundus images in community and in-hospital screening scenarios.

**Methods:**

We collected two color fundus image datasets, namely, PUMCH (556 images, 166 subjects, and four camera models) and NSDE (534 images, 134 subjects, and two camera models). The AI algorithm generates the screening report after taking fundus images. The images were labeled as RDR, RMD, GCS, or none of the three by 3 licensed ophthalmologists. The resulting labels were treated as “ground truth” and then were used to compare against the AI screening reports to validate the sensitivity, specificity, and area under the receiver operating characteristic curve (AUC) of the AI algorithm.

**Results:**

On the PUMCH dataset, regarding the prediction of RDR, the AI algorithm achieved overall results of 0.950 ± 0.058, 0.963 ± 0.024, and 0.954 ± 0.049 on sensitivity, specificity, and AUC, respectively. For RMD, the overall results are 0.919 ± 0.073, 0.929 ± 0.039, and 0.974 ± 0.009. For GCS, the overall results are 0.950 ± 0.059, 0.946 ± 0.016, and 0.976 ± 0.025.

**Conclusion:**

The AI algorithm can work robustly with various fundus camera models and achieve high accuracies for detecting RDR, RMD, and GCS.

## Introduction

Deep learning (DL) has achieved high performance in ophthalmic disease detection based on fundus images, including diabetic retinopathy, glaucoma suspect, and age-related macular degeneration (1). However, there is still a gap between academic research and real-world application. Many DL-based eye disease screening products have suffered major reverses in practice. The reasons for their failures include the low quality of the source fundus images, the difference in distribution between the experimental data and real data, the domain shift caused by different acquisition equipment, and the incapability of detecting multiple eye diseases. Recently, the AI screening algorithm can accurately and robustly detect referable diabetic retinopathy (RDR), referable macular diseases (RMD), and glaucoma suspect (GCS) on fundus images in the experimental environment, which is called AI-100 (2–10). To validate its performance in real-world fundus screening scenarios, we collected data in two typical real-world fundus screening scenarios and made a comprehensive analysis of its performance in these two scenarios.

We selected in-hospital screening and community screening as two typical scenarios. These two scenarios are two extreme situations of eye disease screening. As for the in-hospital scenario, most subjects in the hospitals are follow-up visit patients or suspected patients, thus having a high possibility to be positive cases. In such a scenario, model sensitivity should be high enough to avoid omission. In contrast, in the community screening scenario, there will be a large number of volunteers but most of them will be healthy. Thus, a high model specificity is essential to avoid the waste of medical resources. Since there is always a tradeoff between model sensitivity and specificity, achieving high performance in both of the scenarios is a huge challenge for an automatic eye disease screening algorithm. Therefore, these two scenarios can well–measure the screening algorithm performance in practice.

Besides the different data distribution, another factor affecting the model performance in practice is the multiple diseases. Since some common eye diseases, such as diabetic retinopathy, macular diseases, and glaucoma, share several similar symptoms in their early stages, a patient who accepts diabetic retinopathy screening may actually suffer from macular diseases. However, most existing automated screening models can only detect certain specific diseases. This may cause the neglect of the patients' real diseases, causing the delay in the treatment. Thus, in the in-hospital scenario, we collected the fundus images from a mixture of patients with several different eye diseases, including diabetic retinopathy, macular diseases, and glaucoma, which are those most commonly appeared. This dataset, which contains multiple eye diseases, can well–reflect the model's performance on multiple eye diseases.

Another obstacle in the application of DL-based eye disease detection is domain shift. In practice, different organizations often use different types of cameras to take fundus images. The images taken using different cameras may show a large difference, including the image color, saturation, clarity, and so on (11). Although DL-based methods achieve good performance in eye disease prediction, they are notoriously vulnerable to domain shifts, i.e., the heterogeneous image distribution differences coming from different cameras (12–19). As a result, few DL-based automatic eye disease screening algorithms can be widely applied in the real world. To validate the robustness of the AI-100 algorithm, we collected fundus images from four different cameras in one dataset and from two different cameras in another dataset. As a result, our test results can well–reflect whether the screening algorithm can be popularized in the variable real screening scenarios. The two datasets, namely, the PUMCH dataset and NSDE dataset, are introduced in the next section.

From the two datasets mentioned above, we validated the AI-100 algorithm performance by sensitivity, specificity, and AUC values across different domains of interest. We also compared AI-100 with a strong baseline implemented by ResNet-101 (20), which is one of the most commonly used network architectures. The experiments show that AI-100 can accurately and robustly detect referable diabetic retinopathy (RDR), referable macular diseases (RMD), and glaucoma suspect (GCS) among fundus images of various camera models in both scenarios. Such a result indicates that AI-100 has high application potential in practical eye disease screening programs.

## Materials and methods

### Image acquisition

We collected the fundus image datasets from two typical scenarios, i.e., in-hospital and community screening, to assess the performance of AI-100. We collected a total of 1,090 images from 300 subjects. To further assess the robustness of the screening system, images were taken from various fundus cameras. The studies involving human participants were reviewed and approved by Peking Union Medical College Hospital Review Board. The detailed acquisition process of the datasets is as follows.

The sample size of the study was determined by the significance level, the power of the test, and the estimated and target values of sensitivity and specificity. The formula for calculating the sample size (for each target disease) is as follows:


$$\begin{array}{l} N=\frac{{\left[Z_{1-\alpha }\sqrt{P_{0}\left(1-P_{0}\right)}+Z_{1-\beta }\sqrt{P_{T}\left(1-P_{T}\right)}\right]}^{2}}{{\left(P_{T}-P_{0}\right)}^{2}}, \end{array}$$

where α = 0.05 is the level of significance, 1−β = 0.8 is the power of the test, *P*_0_ is the least acceptable value for sensitivity (or specificity) of the clinical study, and *P*_*T*_ is the estimated sensitivity (or specificity) set in reference to the internal validation results.

(a) PUMCH dataset (in-hospital scenario)

We invited the guests from an education club, which was held by the Department of Ophthalmology at Peking Union Medical College Hospital (PUMCH) on 15 and 16 December 2018 to participate in this study. These guests included the patients, the kinsfolk members of the patients, and the potential patients waiting for screening. The patients had glaucoma, macular, or retinal vascular diseases. The age of participants was between 25 and 86 years (median 63 years). Four fundus cameras of different manufacturers were used for image acquisition. All the participants could freely choose any, some, or all of the four fundus cameras to screen. We used the Topcon TRC-NW400, Syseye RetiCam 3,100, iCare iCare DRS, and Canon CR-2 AF cameras. The detailed specifications of the cameras are shown in Table 1. All the participants signed written informed consent for fundus image acquisition and usage for publication. The study procedures conformed to the tenets of the Declaration of Helsinki, and the study was approved by the Institutional Ethics Committee of the Chinese Academy of Medical Sciences, Peking Union Medical College Hospital (Approval No. S-K1069).

**Table 1:** Technical specifications of different cameras.

**Camera Brand**	**Topcon**	**Syseye**	**iCare**	**Canon**
Type	TRC-NW400	RetiCam 3,100	iCare DRS	CR-2 AF
Mode	Automatic	Automatic	Automatic	Manual
Fixation	Center	Center	Macular	Center
Resolution	1,956 × 1.934	2,656 × 1,992	2,592 × 1,944	5,472 × 3,648
Minimum pupil size	3.3 mm	2.8 mm	3.8 mm	3.3 mm
Field of view	45°	50°	45°	45°

(b) National Sight Day Event (NSDE) dataset (community/population screening scenario)

Employees of Baidu Inc. were invited to voluntarily take fundus images at the screening event held on National Sight Day of China in 2020. All participants had signed a written informed consent before taking fundus images. The participants were aged between 23 and 45 (median 27). Two fundus cameras (Topcon TRC-NW400 and Syseye RetiCam 3100, see Table 1) were used for this event. Images of all participants were taken on both of the cameras.

### Image labeling

For the PUMCH dataset, three ophthalmologists (average ophthalmological experience of 5 years) read all the images independently, to label them as RDR, RMD, GCS, or none of the three (the positive labels are not exclusive) on PC. The criteria for labeling RDR conformed with the international grading guidelines (21, 22), and the detailed criteria for labeling RMD and GCS followed the association standards (23). A simple majority voting determined the final “ground truth” label of each image. The resulting labeled dataset consisted of 556 fundus images from 166 subjects. Refer to Table 2 for detailed distributions of diseases and cameras.

**Table 2:** Distribution of diseases and cameras.

	**PUMCH**				**NSDE**	
	**Topcon**	**Syseye**	**iCare**	**Canon**	**Topcon**	**Syseye**
RDR	10/143	3/150	10/142	4/94	0/667	0/579
RMD	56/97	54/99	56/96	36/62	0/667	0/579
GCS	11/142	9/144	12/140	3/95	0/667	0/579

*Each cell shows the number of images with a positive/negative disease label*.

In the NSDE dataset, we had 534 images from 134 participants. Images of all the participants were taken on both of the cameras. One of the participants was taken with only one eye on both cameras, resulting in 267 pairs of images taken of the same eyes on the same day but on two different cameras. Because the participants were relatively young (< 30 years old) and none of them had reported any acute visual impairment, we only referred those with positive AI screening results to the hospital for a checkup at the event. The final labels of these images were later determined by majority voting by the same ophthalmologists of the PUMCH datasets. The resulting labels were negative for all participants.

### Evaluation criteria

We used three metrics, i.e., sensitivity, specificity, and AUC, to quantitatively compare the disease prediction performance of the AI-100 algorithm and the baseline method. For each of the three diseases, we reported the metrics for each camera individually. We then reported the mean and standard deviation of these values across different cameras to compare the overall performance and robustness. In general, a higher mean value indicates better accuracy, and a smaller standard deviation means better robustness.

We also investigated the 95% CIs of sensitivity, specificity, and AUC. For sensitivity and specificity, we used the exact Clopper-Pearson Cis (24). For the AUC, we used a fast implementation of an existing algorithm to compute the Cis (25). We carried out all the statistical analyses using Python (3.6) with SciPy (1.0.0) and scikit-learn (0.21.3).

Intuitively, a robust model should give close prediction scores on the images of the same eye even if the images are taken by two different cameras. As the NSDE dataset had a pairwise property, we used the mean absolute difference and the Pearson correlation between the prediction scores on different cameras of the same eye to evaluate the model robustness. The mean absolute difference is defined as follows:


$$MAD_{M}=\;\frac{1}{N}\overset{N}{\underset{i=1}{\sum }}|pred_{i}^{A,\;M}-pred_{i}^{B,M}|,\;$$

where *N* is the total number of paired images (in our case, 267), *A* and *B* represent the two different cameras, and *M* is the model of interest (the baseline model or AI-100). The Pearson correlation is computed between the vector $\left[pred_{i}^{A,M}\right]$ and $\left[pred_{i}^{B,M}\right]$, *i* = 1, 2, …*N*. A smaller mean absolute difference or a larger Pearson correlation indicates the model with better robustness.

### The baseline model

To validate the performance of AI-100, we compared it with the ResNet-101 (26) network, which is a commonly used DL model for eye disease detection. For each of the disease prediction tasks, we trained a ResNet-101 (26) network over the AI-100 training dataset. Each disease prediction task is a binary classification problem, supervised by focal loss. The Adam optimizer is used for the gradient descent process, and the learning rates are set to decay from 0.001 to 0.0001. The trained ResNet-101 models were used to predict the three diseases on the PUMCH and NSDE datasets. These predictions were compared against the labels given by the ophthalmologists to get the baseline results.

### AI-100 screening algorithm

**Figure 1:**
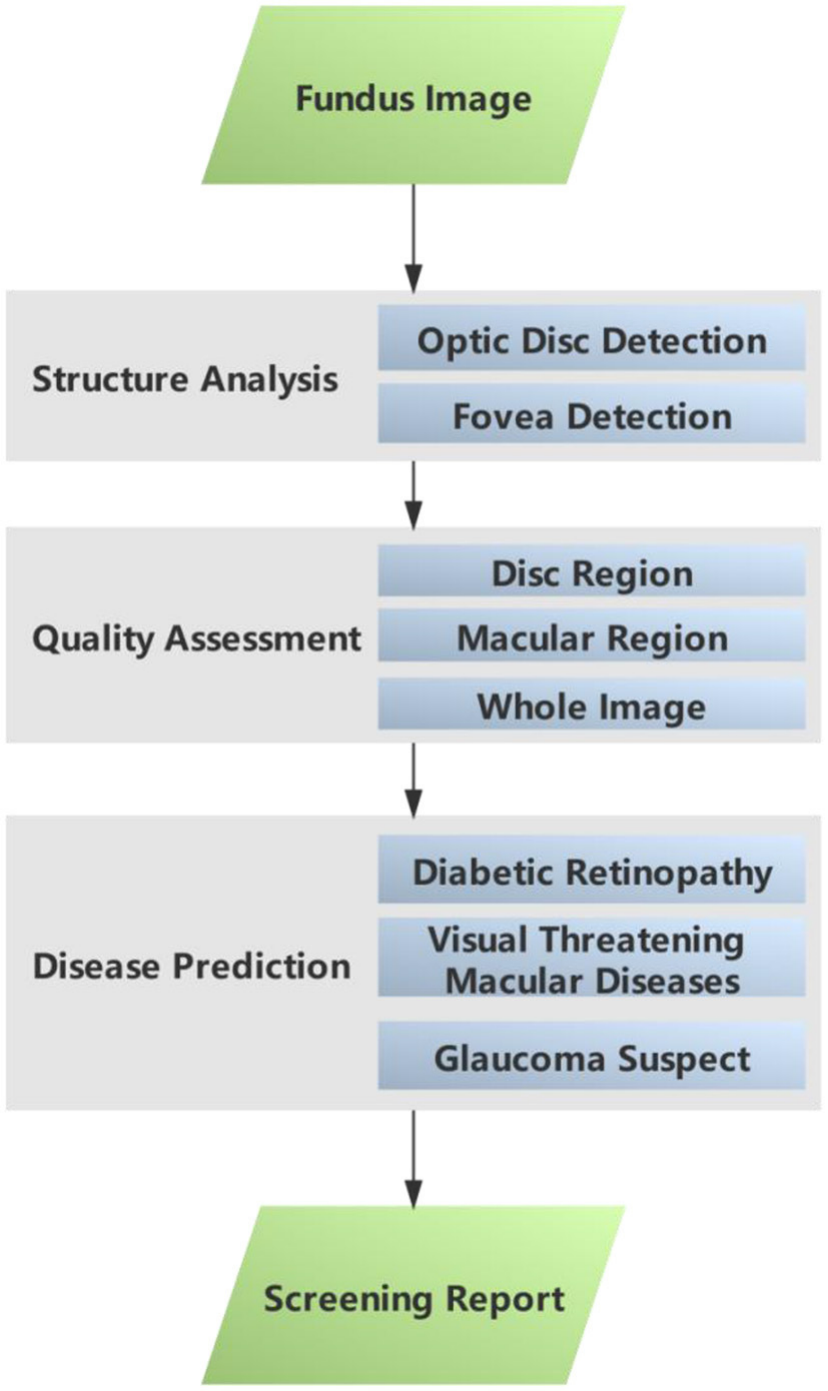
Modules and processes in the AI-100 screening system.

This sub–section gives a brief introduction to the AI-100 algorithm. The AI-100 algorithm consists of three major modules, namely, structural analysis, quality assessment, and disease prediction (refer to Figure 1). In the structural analysis module, the optic disc detection and fovea detection submodules first locate the region of interest (ROI) that is relevant to GCS and RMD, respectively. The quality assessment module decides whether each ROI (as well as the whole image) is of low quality based on a comprehensive analysis of the brightness, contrast, and blurriness. If any of the ROIs is determined as low quality, the image is disqualified from the study and the system prompts for a retake. The disease prediction module consists of three self-designed deep learning models, which are designed to predict RDR, RMD, and GCS, respectively. The three models share a similar backbone architecture.

The backbone of the disease prediction module is designed as a combination of densenet-121 (27) and bilinear pooling. Detailed architecture is shown in Table 3. This structure alleviates the gradient disappearance phenomenon during model training by stacking multiple dense blocks with connections and strengthens the internal feature reuse of the model to achieve high accuracy. In addition, to obtain a more accurate prediction model, AI-100 adopts the following strategies to improve the model:

**Table 3:** The network backbone of the AI-100 algorithm.

**Layer**	**Output size**	**Architecture**
Convolution	112 × 112	7 × 7 *conv, stride* 2
Pooling	56 × 56	3 × 3 max *pooling, stride* 2
Dense Block (1)	56 × 56	
Transition Layer (1)	56 × 56	1 × 1 *conv*
	28 × 28	2 × 2 *average pool, stride* 2
Dense Block (2)	28 × 28	
Transition Layer (2)	28 × 28	1 × 1 *conv*
	14 × 14	2 × 2 *average pool, stride* 2
Dense Block (3)	14 × 14	
Transition Layer (3)	14 × 14	1 × 1 *conv*
	7 × 7	2 × 2 *average pool, stride* 2
Dense Block (4)	7 × 7	
Classification Layer	1 × 1	7 × 7 *global average pool*
		1000*D fully*−*connected*, *softmax*

(a) The input image size is increased from 224 × 224 to 512 × 512, which helps to better preserve the detailed information of the original image.

(b) The bilinear pooling layer is used to replace the gap layer in the original densenet-121. As a general technique in the field of fine-grained image classification, the bilinear pooling layer can help the model extract the texture information in the image (such as diabetic retinopathy-related bleeding/specific signs) to help the model focus on discriminative features.

(c) For diabetic retinopathy detection, the model will output fine-grained grading results (no diabetic retinopathy/phase I/phase II/phase III/phase IV or above), and then transform it into binary/ternary classification results through probability weighting. When modeling and optimizing the model, AI-100 encodes the classification label by ordinal regression. Compared to one-hot labels, ordinal regression labels can better model the semantic relationship between diabetic retinopathy grades, so that the distance between the labels of similar grades will be closer, while the distance between labels with a large span will be relatively larger.

**Figure 2:**
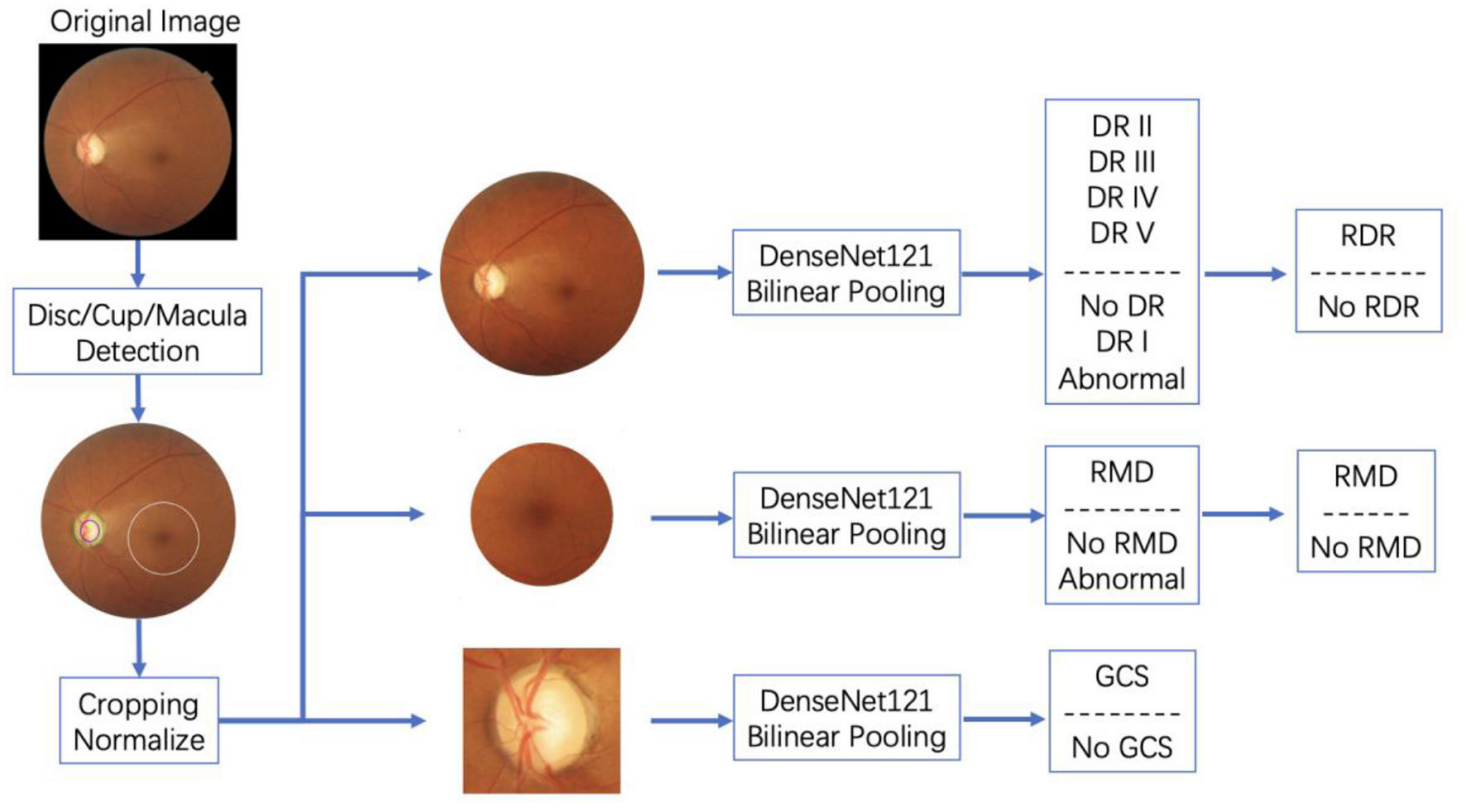
Details of disease prediction modules in AI-100.

In addition, in order to better distinguish the patients with the target disease and those with other ophthalmic diseases but not the target one, AI-100 adds an additional category, named “abnormal”, in the model to reduce the intra-class divergence of the model during training. An illustration of how AI-100 works on fundus images is shown in Figure 2.

The AI-100 system was trained on the dataset with 52,405 samples in total. The training dataset contained 4,865 cases of RDR, 16,672 cases of RMD, and 3,225 cases of GCS. In that, 12,923 images were sampled from camera Canon CR-2 AF, 33,480 images were sampled from camera Kowa VX-10i, 3,938 images were sampled from camera Zeiss VISUCAM 200, and 2,064 images were sampled from other cameras. The disc/cup/macula detection module and the three disease classification modules were, respectively, trained on this dataset. In the disc/cup/macula detection module, faster-RCNN was first trained to locate the optic disc region. Then a segmentation model was used to extract the optic cup out of the optic disc region. The macular area of interest (AOI) was calculated based on the relative position between the fovea and the optic disc. After the optic disc, optic cup, and macular AOI were detected, the raw images were cropped to the region of interest and sent to the three disease classification models, respectively. The three classification models were then trained in an end-to-end manner to predict the probability of the target disease. The standard cross-entropy loss was adopted for the back-propagation. A more detailed training process for AI-100 can be found in the book chapter (10).

The AI-100 system was run locally on a desktop computer (Intel I5-8,400 and NVIDIA GeForce GTX 1,060), and the participants received their printed AI screening reports within 30 s after taking the images (including report printing). An AI screening report suggests if the participant is positive for RDR, RMD, and GCS or not. We also recorded the prediction value of each disease internally to construct the ROC curve later. The results were compared against the labels given by ophthalmologists to evaluate the performance.

## Results

### Performance of disease prediction using the baseline model on the PUMCH dataset

Table 4 presents the performance of the baseline model (ResNet-101) in predicting RDR, RMD, and GCS across four camera brands on the PUMCH dataset. For most of the tasks, the baseline model can achieve sensitivity and specificity values > 0.8. For the prediction of RDR, the baseline model achieves good AUCs (0.952 and 0.949) on Topcon and Syseye cameras but relatively poor AUCs (0.818 and 0.822) on iCare and Canon cameras. For the prediction of RMD, the baseline model achieves high AUCs (0.964, 0.930, and 0.938) on Topcon, Syseye, and iCare cameras but a relatively low AUC of 0.887 on Canon cameras. For the prediction of GCS, the baseline model achieves high AUCs (0.964 and 0.982) on Topcon and Canon cameras but relatively low AUCs (0.803 and 0.903) on Syseye and iCare cameras.

**Table 4:** Prediction performance of the baseline model on RDR, RMD, and GCS across cameras.

	**Camera brand**	**Topcon**	**Syseye**	**iCare**	**Canon**
RDR	Sensitivity	1.000 (0.692, 1.000)	1.000 (0.439, 1.000)	0.700 (0.348, 0.933)	0.500 (0.068, 0.932)
	Specificity	0.762 (0.684, 0.829)	0.767 (0.691, 0.832)	0.852 (0.783, 0.906)	0.915 (0.839, 0.963)
	AUC	0.952 (0.902, 1.000)	0.949 (0.902, 0.996)	0.818 (0.646, 0.989)	0.822 (0.601, 1.000)
RMD	Sensitivity	0.821 (0.696, 0.911)	0.870 (0.751, 0.946)	0.857 (0.738, 0.936)	0.861 (0.705, 0.953)
	Specificity	0.928 (0.857,0.971)	0.758 (0.661, 0.838)	0.865 (0.780,0.926)	0.758 (0.633, 0.858)
	AUC	0.964 (0.937, 0.992)	0.930 (0.886, 0.973)	0.938 (0.901, 0.975)	0.887 (0.819, 0.955)
GCS	Sensitivity	0.909 (0.587, 0.998)	0.556 (0.212, 0.863)	0.833 (0.516, 0.979)	1.000 (0.292, 1.000)
	Specificity	0.866 (0.799, 0.918)	0.861 (0.794, 0.913)	0.843 (0.772, 0.899)	0.842 (0.753, 0.909)
	AUC	0.964 (0.927, 1.000)	0.803 (0.633, 0.974)	0.903 (0.788, 1.000)	0.982 (0.952, 1.000)

*The numbers in the parentheses are lower and upper bounds of the respective 95% CIs*.

### Performance of disease prediction using AI-100 on PUMCH dataset

Table 5 provides the performance of AI-100 in predicting the three types of diseases across four camera brands. Notably, the sensitivity and specificity values are higher than 0.85 and the AUC values are > 0.9 for all the tasks across all camera models.

**Table 5:** Prediction performance of AI-100 on RDR, RMD, and GCS across cameras.

	**Camera brand**	**Topcon**	**Syseye**	**iCare**	**Canon**
RDR	Sensitivity	0.900 (0.555, 0.998)	1.000 (0.292, 1.000)	0.900 (0.555, 0.998)	1.000 (0.398, 1.000)
	Specificity	0.930 (0.875, 0.966)	0.967 (0.924, 0.989)	0.986 (0.950, 0.998)	0.968 (0.910,0.993)
	AUC	0.919 (0.781, 1.000)	1.000 (0.999, 1.000)	0.906 (0.721, 1.000)	0.992 (0.976, 1.000)
RMD	Sensitivity	0.982 (0.905, 1.000)	0.852 (0.729, 0.934)	0.982 (0.905, 1.000)	0.861 (0.705, 0.953)
	Specificity	0.907 (0.831,0.957)	0.970 (0.914,0.994)	0.885 (0.804, 0.941)	0.952 (0.865, 0.990)
	AUC	0.978 (0.956, 1.000)	0.966 (0.939, 0.993)	0.985 (0.972, 0.999)	0.968 (0.937, 1.000)
GCS	Sensitivity	0.909 (0.587, 0.998)	0.889 (0.518, 0.997)	1.000 (0.735, 1.000)	1.000 (0.292, 1.000)
	Specificity	0.944 (0.892, 0.975)	0.924 (0.867, 0.961)	0.957 (0.909, 0.984)	0.958 (0.896, 0.988)
	AUC	0.941 (0.826, 1.000)	0.973 (0.932, 1.000)	0.996 (0.989, 1.000)	0.993 (0.976, 1.000)

*The numbers in the parentheses are lower and upper bounds of the respective 95% CIs*.

### Performance and robustness comparison between the baseline model and AI-100

To compare both the performance and robustness, we compute the mean values and standard deviations of sensitivity, specificity, and AUCs over different cameras, for RDR, RMD, and GCS prediction, respectively. The results are shown in Table 6. A higher mean value denotes better overall performance, and a lower standard deviation denotes better robustness to cameras. In terms of performance, AI-100 is better than the baseline model in predicting all three diseases. In terms of robustness, AI-100 outperforms the baseline model on all items except for the sensitivity of RMD and the specificity of GCS prediction.

**Table 6:** Mean and standard deviation of performance values for AI-100 and the baseline model, respectively.

		**AI-100**	**Baseline**
RDR	sensitivity	0.950 ± 0.058	0.800 ± 0.245
	specificity	0.963 ± 0.024	0.824 ± 0.073
	AUC	0.954 ± 0.049	0.885 ± 0.075
RMD	sensitivity	0.919 ± 0.073	0.852 ± 0.022
	specificity	0.929 ± 0.039	0.827 ± 0.084
	AUC	0.974 ± 0.009	0.930 ± 0.032
GCS	sensitivity	0.950 ± 0.059	0.825 ± 0.192
	specificity	0.946 ± 0.016	0.853 ± 0.012
	AUC	0.976 ± 0.025	0.913 ± 0.081

### Performance and robustness comparison on NSDE dataset

Since the NSDE dataset does not have positive labels, we report only the specificity across two cameras for performance comparison (Table 7). For robustness comparison, the mean absolute difference and the Pearson correlation are shown in Table 8. Given the 267 data points, all the differences between the two values in each row of Table 8 are significant (*p*-values < 0.0001).

**Table 7:** Specificity values of AI-100 and baseline models on Topcon and Syseye with NSDE data.

	**Model**	**Baseline**	**AI-100**	**Baseline**	**AI-100**
	**Camera**	**Topcon**	**Topcon**	**Syseye**	**Syseye**
RDR	Specificity	0.944 (0.909, 0.968)	0.981 (0.957, 0.994)	0.918 (0.878,0.948)	0.963 (0.932, 0.982)
RMD	Specificity	0.955 (0.923, 0.977)	0.966 (0.937,0.985)	0.843 (0.793, 0.884)	0.959 (0.928,0.979)
GCS	Specificity	0.963 (0.982, 0.932)	0.974 (0.947, 0.989)	0.921 (0.882, 0.951)	0.996 (0.979, 0.999)

*The numbers in the parentheses are lower and upper bounds of the respective 95% CIs*.

**Table 8:** Mean absolute difference and Pearson correlation of prediction scores on pairwise images of same eye and different cameras.

		**Baseline**	**AI-100**
RDR	MAD (mean ± std.)	0.057 ± 0.115	0.032 ± 0.023
	correlation	0.295	0.739
RMD	MAD (mean ± std.)	0.095 ± 0.103	0.036 ± 0.029
	Correlation	0.231	0.922
GCS	MAD (mean ± std.)	0.073 ± 0.121	0.022 ± 0.030
	Correlation	0.229	0.776

### Performance on PUMCH-NSDE mixed dataset

Since the NSDE dataset does not have positive labels, the model sensitivity in this scenario has not been well–validated. Thus, we supply a PUMCH-NSDE mixed dataset to validate the model's performance more comprehensively. The mixed dataset resulted from the stratified sampling of PUMCH and NSDE datasets with a division of age 40, which ensures the median age of the patients is 40 (close to the current census median age). The results are shown in Table 9. The AI-100 still shows high performance on the PUMCH-NSDE mixed dataset, which verifies its capability in the community scenario.

**Table 9:** Specificity values of AI-100 on Topcon and Syseye with PUMCH-NSDE mixed data.

	**Camera**	**Topcon**	**Syseye**
RDR	Sensitivity	0.889 (0.518, 0.997)	1.000 (0.292, 1.000)
	Specificity	0.961 (0.907, 0.990)	0.960 (0.902, 0.989)
RMD	Sensitivity	0.963 (0.935, 0.982)	0.833 (0.717, 0.921)
	Specificity	0.971 (0.919, 0.994)	0.959 (0.898, 0.989)
GCS	Sensitivity	0.918 (0.615, 0.998)	0.875 (0.474, 0.997)
	Specificity	0.963 (0.908, 0.990)	0.982 (0.935, 0.998)

*The numbers in the parentheses are lower and upper bounds of the respective 95% CIs*.

## Discussion

The main purpose of the study is to validate the practicability of the AI-100 automated eye disease screening algorithm. Toward that end, we tested the performance of AI-100 in two typical eye disease screening scenarios. The robustness was also tested by taking images from various cameras. The results show that AI-100 can detect RDR, RMD, and GCS with high sensitivity and specificity and low variance among different cameras.

We highlight that this study is prospective since the images are taken from where the AI-100 is fixed and deployed. In general, in retrospective studies, the training image and the testing image are randomly sampled from a unified patient cohort, thus the training set and the testing set will likely have the same distribution. As a result, the model's robustness and generalization ability cannot be well-reflected.

The AI-100 shows an overall better performance than the baseline model in all four camera brands, three diseases, and two population groups. The fluctuations of AI-100 performance on different camera models are generally smaller than the baseline model, according to the standard deviation comparison in Table 6. In the PUMCH dataset, the AUC standard deviation of AI-100 among cameras is 0.049, 0.009, and 0.025 for RDR, RMD, and GCS, respectively, while the corresponding results of the baseline method are 0.075, 0.032, and 0.081.

Although AI-100 gives a robust performance in terms of AUC, it still exhibits some levels of fluctuations in terms of sensitivity and specificity. For example, the sensitivity of the AI-100 prediction RMD has a standard deviation of 0.073, suggesting that a customized decision threshold according to a specific camera model may help to achieve a better performance balance.

In the PUMCH dataset, the participants have already visited the department of ophthalmology, thus there is a fair number of positive patients. We notice that some camera models have very few positive cases (e.g., the brand Syseye has only three positive images for RDR, refer to Table 2), resulting in a larger confidence interval and undermining the statistical power of the corresponding result. In the NSDE dataset, none of the participants is positive for any of the three diseases. As a result, approximately 10 participants made an unnecessary visit to the hospital (some of the participants were confirmed negative through their annual physical examination). This reflects a common challenge of false positives in a screening event where the prevalence of the target disease is extremely low.

In the analysis of false positives and false negatives examples, we find most false positives are due to the interference of other diseases or lesions. For example, the prediction of RDR interferes with hypertension fundus, venous obstruction, retinitis pigmentosa, minor hemorrhages, etc. One way to solve this may be to train a more comprehensive AI model to discriminate against these diseases/lesions. Another possible solution is to combine some other medical records and exams to achieve more reliable predictions. Some other false positives are caused by technical problems, e.g., the stains on the camera lens and the limited field of view. However, these technical pitfalls cannot be avoided in real-world use, especially in primary care. In the future, we may introduce the neural network interpretation (28) methods for further analysis.

The high specificity of the NSDE and PUMCH-NSDE mixed datasets suggests that AI-100 is suitable for screening purposes. In the community screening scenario, high specificity is essential. Generally, a large number of people go for the free community screening, whereas the percentage of real patients is actually very low (27). Low specificity will lead to a huge waste of medical resources. The AI-100 has a specificity of more than 96% on the NSDE dataset (mostly healthy people) and therefore is applicable to the community screening scenario.

In summary, we investigate the feasibility of using an AI algorithm, called AI-100, to screen for RDR, RMD, and GCS on fundus images captured by four different camera models. AI algorithm shows high sensitivity and specificity for all diseases, and the performance fluctuations are acceptable among camera models. As such, the AI-100 screening algorithm has value for RDR, RMD, and GCS screening and can potentially be applied to existing fundus cameras of various manufacturers.

## Data availability statement

The raw data supporting the conclusions of this article will be made available by the authors, without undue reservation.

## Ethics statement

The studies involving human participants were reviewed and approved by Peking Union Medical College Hospital Review Board. The patients/participants provided their written informed consent to participate in this study.

## Author contributions

RH, GC, BZ, JY, and MY collected, annotated the data, and designed the experiments. CZ, YC, and YX led the project. DY, JW, and JL conducted the experiments and analyzed the results. All authors contributed to the article and approved the submitted version.

## Conflict of interest

The authors declare that the research was conducted in the absence of any commercial or financial relationships that could be construed as a potential conflict of interest.

## Publisher's note

All claims expressed in this article are solely those of the authors and do not necessarily represent those of their affiliated organizations, or those of the publisher, the editors and the reviewers. Any product that may be evaluated in this article, or claim that may be made by its manufacturer, is not guaranteed or endorsed by the publisher.

## Abbreviations

RDR: referable diabetic retinopathy
RMD: referable macular diseases
GCS: glaucoma suspect
CI: confidence interval
AUC: area under the receiver operating characteristic curve
